# Peptides Corresponding to the Predicted Heptad Repeat 2 Domain of the Feline Coronavirus Spike Protein Are Potent Inhibitors of Viral Infection

**DOI:** 10.1371/journal.pone.0082081

**Published:** 2013-12-03

**Authors:** I-Jung Liu, Wan-Ting Tsai, Li-En Hsieh, Ling-Ling Chueh

**Affiliations:** 1 Department of Nursing, Cardinal Tien Junior College of Healthcare and Management, New Taipei City, Taiwan; 2 Graduate Institute of Veterinary Medicine, School of Veterinary Medicine, National Taiwan University, Taipei, Taiwan; The University of Hong Kong, Hong Kong

## Abstract

**Background:**

Feline infectious peritonitis (FIP) is a lethal immune-mediated disease caused by feline coronavirus (FCoV). Currently, no therapy with proven efficacy is available. In searching for agents that may prove clinically effective against FCoV infection, five analogous overlapping peptides were designed and synthesized based on the putative heptad repeat 2 (HR2) sequence of the spike protein of FCoV, and the antiviral efficacy was evaluated.

**Methods:**

Plaque reduction assay and MTT (3-(4,5-dimethylthiazol-2-yl)-2,5-diphenyltetrazolium bromide) cytotoxicity assay were performed in this study. Peptides were selected using a plaque reduction assay to inhibit Feline coronavirus infection.

**Results:**

The results demonstrated that peptide (FP5) at concentrations below 20 μM inhibited viral replication by up to 97%. The peptide (FP5) exhibiting the most effective antiviral effect was further combined with a known anti-viral agent, human interferon-α (IFN-α), and a significant synergistic antiviral effect was observed.

**Conclusion:**

Our data suggest that the synthetic peptide FP5 could serve as a valuable addition to the current FIP prevention methods.

## Introduction

Feline coronavirus (FCoV) is a group 1a coronavirus [1] that usually causes mild gastrointestinal symptoms in cats; however, a small percentage of seropositive animals (5 to 12%) can develop highly lethal Feline infectious peritonitis (FIP). Neither an effective vaccine nor any effective drugs are currently available for the prevention and control of this disease [2]. Interferon (IFN), immune inhibitors, immune modulators and supportive therapy have been used in clinics, but their efficacy has typically been poor [3,4]. 

Since the pandemic of severe acute respiratory syndrome (SARS) in 2003, numerous antiviral drugs have been developed to control SARS coronavirus (SARS-CoV), including S protein heptad repeat (HR) peptides, carbohydrate-binding agents, cathepsin inhibitors, HIV protease inhibitors, nitric oxide, siRNA and interferons [5]. Except for the S protein HR peptides, all the above-mentioned reagents have been tested for their ability to inhibit FCoV replication [6-11], e.g., Cathepsin L and cathepsin B can be inhibitors to inhibit the entry of FIPV-1146 [9] and pyridine *N*-oxide derivatives were found inhibitory against coronaviruses, in particular against the feline coronavirus type II strain of FIPV[11].

The S protein of FCoV consists of the S1 and S2 subunits. S1 is located at the N-terminus of the molecule and has a spherical structure that includes the domain that binds to receptors on the host cell [12]. S2 is located at the C-terminus of the spike protein and contains HR1 and HR2, which are the primary regions responsible for the fusion of the virus to the cell membrane of the host. Upon the binding of S1 to the host receptor, S2 undergoes a structural transformation that exposes HR1 and HR2, which form a six-helix bundle structure [13,14]. This type of helix structure formation is characteristic of class I viral fusion [15]. Many in vitro studies of SARS-CoV have reported that exogenous HR2 peptide can bind to viral HR1, thereby efficiently blocking the entry of the virus into cells and inhibiting the replication of the virus [16-19].

Multiple agents that target different stages of viral replication have been successfully used to control infections in humans, such as Human immunodeficiency virus (HIV) and Human hepatitis C virus (HCV) [20-22]. With regard to FCoV infection, our previous in vitro study demonstrated that the combination of nelfinavir and *Galanthus nivalis* agglutinin (GNA) has a synergistic antiviral effect that inhibits FCoV replication [6]. In order to find a less expensive and more effective antiviral combination to improve the prognosis of cats with FIP, peptides based on the putative HR2 of the S protein were designed and tested for their inhibitory effect, and the possible synergism of these peptides with other known anti-FCoV agent was investigated. 

## Materials and Methods

### Cell and virus

*Felis catus* whole fetus-4 (fcwf-4) cells [23], [24] (kindly provided by Professor Peter J. M. Rottier, Utrecht University) were maintained in Dulbecco’s modified Eagle’s medium (DMEM) supplemented with 5% fetal bovine serum (FBS), 100 IU/mL penicillin and 100 μg/mL streptomycin in 5% CO_2_ at 37°C. The type II NTU156 strain of the virus was isolated locally [25].

### Peptides and MTT cytotoxicity assay for peptides

**Figure 1:**
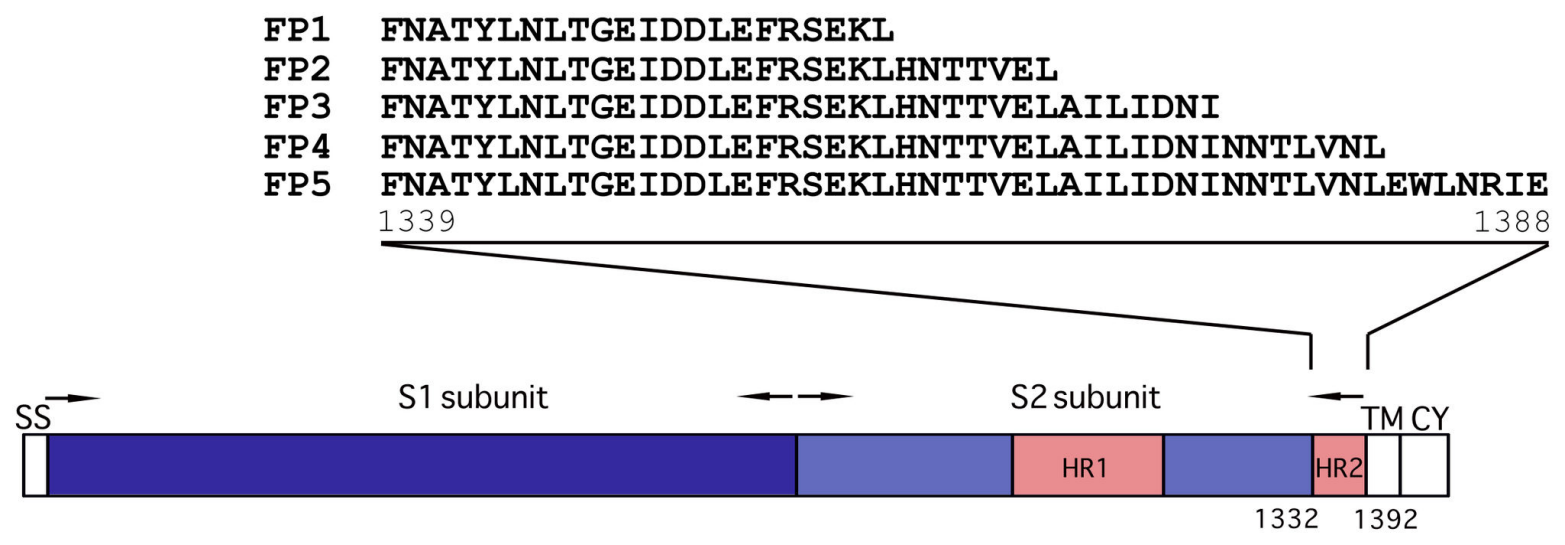
Schematic drawing of S protein of FCoV (NTU156) and the designed peptides. The S2 subunit contains two heptad repeats (HR1, HR2). The amino acid sequences of peptides (FP1-FP5) in HR2 are shown. ss, signal sequence. TM, transmembrane domain. CY, cytoplasmic tail. The numbers of amino acid residues are indicated.

The sequences of the FCoV S protein HR peptides (FCoV HR peptides) were designed based on the S protein of type II FCoV NTU156 (GenBank accession no. ACS44218.1), and all the peptides were dissolved in aqueous NaOH (pH 11.3), the aqueous NaOH were prepared from duble distilled water adjusted to pH 11.3 by 1 N NaOH. The peptide sequences are shown in Figure 1. To determine the cytotoxicity of each peptide, fcwf-4 cells (3 ×10^4^ cells/mL) were grown in 96-well plates for 24 h. The cells were then pre-incubated with or without different concentrations of peptides in triplicate at 37°C for 72 h and assessed with the MTT (3-(4,5-dimethylthiazol-2-yl)-2,5-diphenyltetrazolium bromide) assay. Briefly, the cells in each well were incubated with MTT reagent (10 mg/mL) at 37°C for 4 h. The cells were then lysed with lysis buffer (100 μl of 10% SDS, 45% dimethyl formamide, adjusted to pH 4.5 by glacial acid) after removing the MTT. The absorbance value at 570 nm was measured with a microELISA reader. The cell viability was the relative absorbance at 570 nm of samples with peptides relative to the absorbance for samples without peptides. 

### Screening of effective peptides

The effective peptides were selected using a plaque reduction assay. Fcwf-4 cells (5 ×10^4^ cells/mL) were seeded in 48-well plates and incubated at 37°C for 24 h prior to use. Various concentrations of peptides were incubated with NTU156 at a multiplicity of infection (MOI) of 0.1. After incubation for 1 h, the mixtures of peptide and virus were incubated with fcwf-4 cells for 1 h. Then, the supernatants were removed, and DMEM containing 2% FBS was added to each well. The supernatants were collected 48 h postinfection and incubated with fcwf-4 cells. After 1 h of incubation, the supernatants were removed, and DMEM containing 2% FBS was added. The cells were fixed and stained at 72 h postinfection, and the extent of the cytopathic effect (CPE) was assessed. Based on these results, the fifty percent inhibitory concentrations (IC_50_) were calculated using an interpolation method.

### Combination of effective agents against FCoV

FCoV HR peptides were combined with human interferon-alpha (IFN-α) (Roche). IFN-α was preincubated with cells cultured in DMEM containing 2% FBS for 24 h. The supernatants were removed, and the cells were infected with NTU156 at an MOI of 0.1. The supernatants were removed after 1 h of incubation. Peptides at the selected concentrations were incubated with cells in DMEM containing 2% FBS. The supernatants were collected after 48 h of incubation for titration, and the viral titer in the presence of each combination of peptide/IFN-α was determined using a plaque reduction assay as previously described [6]. All samples were analyzed in triplicate. Statistical analyses of the synergism of the antiviral activity were performed using a two-way analysis of variance.

## Results

### HR2 peptides are non-cytotoxic to fcwf-4 cells

To assess the toxicity of the five FCoV HR2 peptides designed in this study, six concentrations of each peptide, ranging from 6.25 μM to 200 μM, were used to treat fcwf-4 cells for 72 h, and the viability of the cells was evaluated. Under the tested concentrations, the cell survival rates were all greater than 95%, indicating that the peptides are not toxic to the host cells with the CC_50_ value ≧200 μM. 

### Inhibition of FCoV replication by HR2 peptides

**Figure 2:**
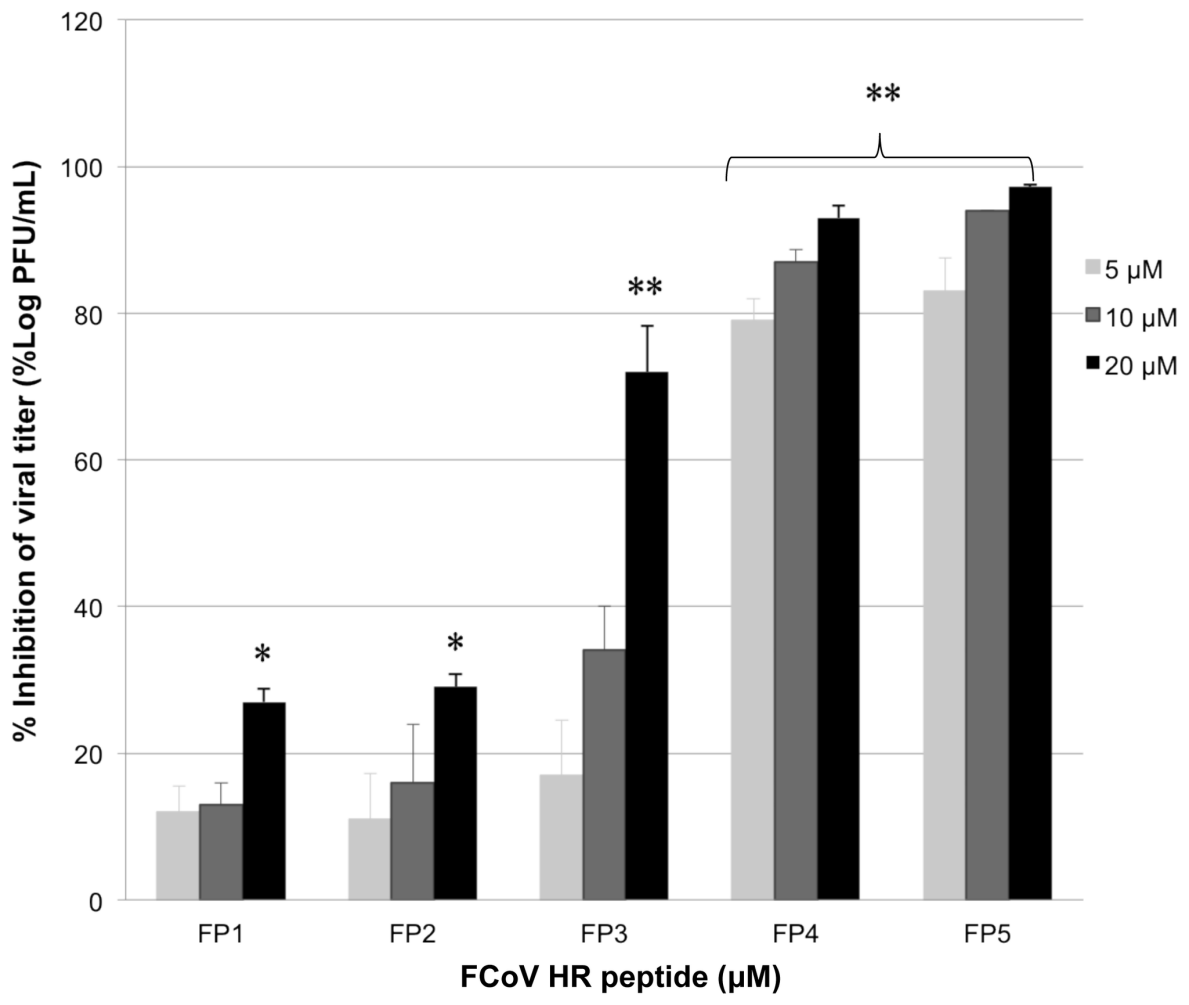
Inhibition of feline coronavirus replication by FCoV HR peptides. The data are the means±standard errors of triplicate wells. One representative experiment of more than two experiments is shown. * indicates significant inhibition of viral replication (P<0.005), ** indicates extremely significant inhibition of viral replication (P<0.0001).

Based on the cell viability results, three concentrations of peptide, i.e., 5 μM, 10 μM, and 20 μM, were chosen for further testing of the peptide’s antiviral effects. The results showed that among the five peptides, only FP4 and FP5 could significantly reduce the viral titer, with reductions of 93% and 97%, respectively (Figure 2). This inhibition was concentration dependent compared with untreated groups from the Table 1 (P<0.0001) (Figure 2). The IC_50_ values of FP4 and FP5 were 1.8 μM and 1.33 μM, respectively (Table 1). FP5 was the most potent inhibitor of FCoV replication among the tested peptides.

**Table 1:** Inhibitory effects of the FCoV HR peptides.

Peptides	Highest concentration tested (μM)	IC_50_ (μM)	CC_50_ (μM)	Selectivity index (SI)	Inhibition of viral titer (%)
FP1	20	NA	≧200	NA	27
FP2	20	NA	≧200	NA	29
FP3	20	14.21	≧200	≧14.07	72
FP4	20	1.8	≧200	≧111.11	93
FP5	20	1.33	≧200	≧150.37	97.2

NA: not analyzed

### Synergistic effect of FCoV HR peptide with human IFN-α

**Figure 3:**
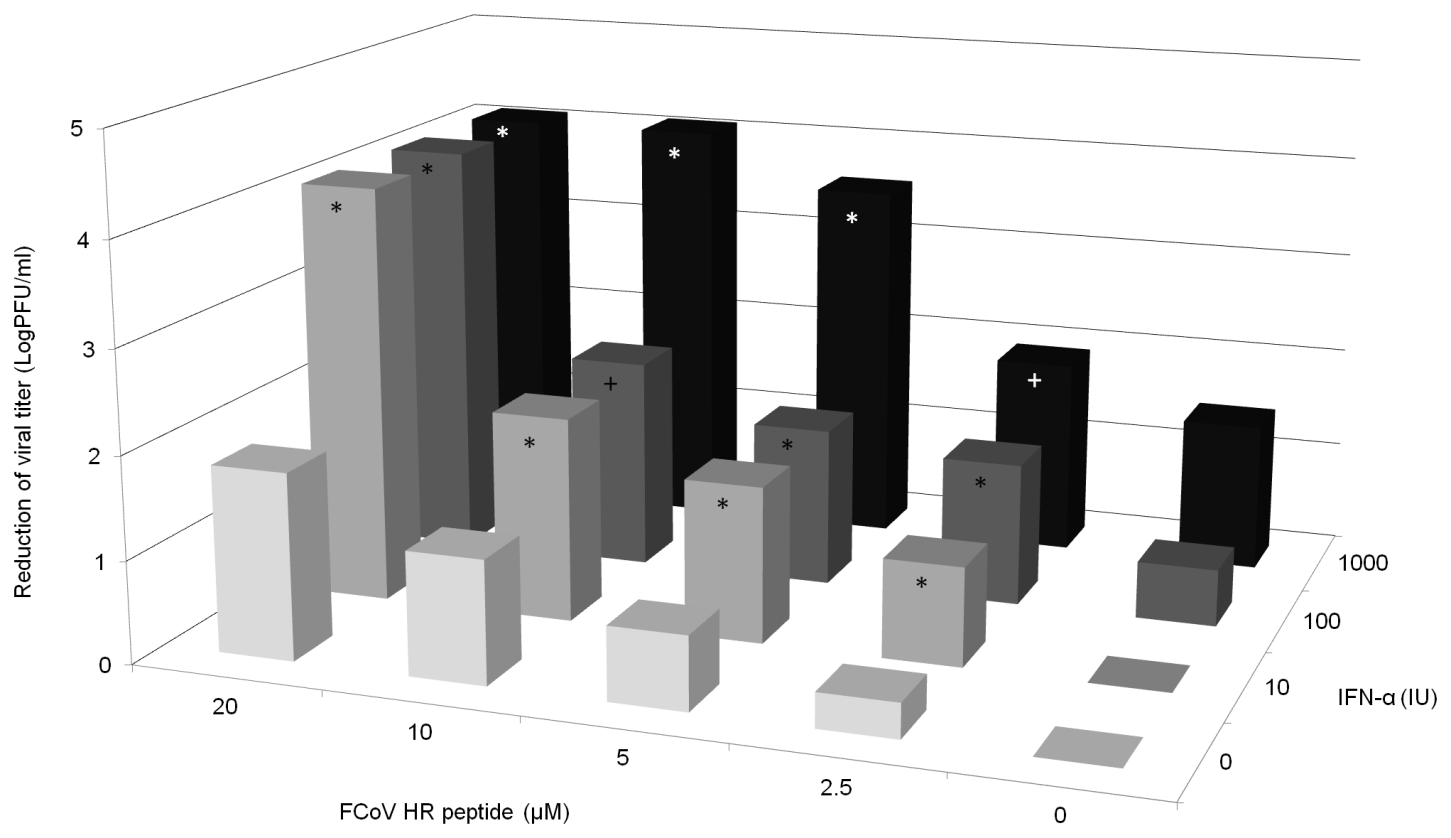
Inhibitory effects of the combination of the FP5 peptide and IFN-α at various concentrations. The data are the means±standard errors of triplicate wells. One representative experiment of more than two experiments is shown. Statistical analyses of the synergism of the antiviral effects were performed using a two-way analysis of variance. * indicate significant synergistic antiviral effect (P<0.05), + indicate additive antiviral effect.

Human IFN-α has been recommended as therapy for FIP. However, the use of IFN-α alone cannot completely control viral replication in cats.  To determine whether FP5, the most effective inhibitor among the FCoV HR peptides, could increase the anti-viral activity when combined with human IFN-α, an inhibition assay was performed (Figure 3). The results showed that when high concentrations of FP5 20 μM and IFN-α 1000 IU were used alone, viral titer reduced by 1.81 and 1.46 LogPFU/ml, respectively, the growth of FCoV could not be blocked completely. However, when 20 μM FP5 was combined with 10 to 1000 IU of IFN-α and when 10 μM FP5 was combined with 1000 IU of IFN-α, a complete inhibitory effect was observed, and viral titer reduced by up to 4.08 LogPFU/ml. This inhibitory effect is higher than the additive effect mediated by the two agents alone (Figure 3). These combinations showed synergetic effects, as determined by a two-way analysis of variance (P < 0.05). 

## Discussion

The utilization of HR peptides derived from spike proteins to interfere the fusion of the viral envelope with host membrane, thus inhibiting viral replication, has been well-known to successfully inhibit the infection of numerous viruses with class I fusion proteins [16,17,26-34]. Among these peptides, the anti-HIV-1 peptide, enfuvirtide (T-20), has already been incorporated into a clinical regimen and shown strong efficacy against HIV infection [35-37]. Although the crystal structure of the S protein of FCoV remains to be determined, by aligning the amino acid sequences derived from FCoV with other members of coronaviruses [38], the HR2 domain was predicted, and peptides corresponding to this domain were designed. HR2 was chosen because it was found to be more effective than HR1 in previous studies [26,31,39]. 

It has been reported that several peptides derived from HR2 domain are able to inhibit SARS-CoV infection with IC_50_ less than 19 µM [18,19,40]. The inhibitory efficacy is not correlated with the lengths of these reported HR2 peptides and the central helix region of HR2 containing amino acid residue 1161 to 1175 are critical to inhibit SARS-CoV infection. In addition, truncation of N-terminal was found to be more related to the loss of potency than the C-terminal [16,19]. While examing the inhibitory effect of our feline peptides we found that FP4 with the extra residures NNTLVNL at its C-terminus compared to FP3, showed a significant potential to inhibit viral replication (Figure 2). The finding indicates that these residues might play an important role in the process of membrane fusion, thus inhibiting viral replication.

Human IFN-α has been reported to have anti-FCoV effects and inhibit viral replication [7]. Synergistic effects were found when IFN-α was combined with ribavirin [40]. However, due to severe adverse reactions to ribavirin, this drug is not feasible for clinical use [41]. The present study found that the combination of the nontoxic peptide FP5 with human IFN-α exhibits a satisfactory synergistic antiviral effect: 10 units of IFN-α combined with 20 μM FP5 resulted in complete inhibition.

One anti-viral HR2 peptide that is currently used for HIV treatment (enfuvirtide, T-20) was shown to cause pain or discomfort at the injection site [42], and other reactions, including tuberous nodules, erythematosus, diarrhea, regurgitation, and pruritus, have been reported [43]. The HR peptide of FCoV must be tested in a follow-up clinical study to determine whether it has adverse effects on felines. 

Because FIP is an immune-mediated disease, in addition to anti-viral drugs to control viral replication, the treatment of this disease also requires IFN, immune inhibitors, or cytokine antagonists to achieve a better prognosis. This study utilized in vitro experiments to demonstrate that an HR peptide can inhibit FCoV replication. Furthermore, when this peptide was combined with human IFN-α, lower doses of both agents were needed to achieve the complete inhibition of viral replication. Our results suggest that the combined use of these two drugs together with appropriate agents to modify immune function is a potential therapy for the treatment of FIP.

## Conclusions

We demonstrated in this study, that the synthetic peptide FP5 could inhibit viral replication. This peptide exhibited a significant synergistic antiviral effect when combined with IFN-α. Our data suggest that combination of the two agents could serve as a valuable addition in the treatment and prevent of FIP. 

## References

[1] Carstens EB (2010) Ratification vote on taxonomic proposals to the International Committee on Taxonomy of Viruses (2009). Arch Virol 155: 133-146. doi:10.1007/s00705-009-0547-x. PubMed: 19960211. 19960211 PMC7086975

[2] Fehr D, Holznagel E, Bolla S, Hauser B, Herrewegh AA et al. (1997) Placebo-controlled evaluation of a modified life virus vaccine against feline infectious peritonitis: safety and efficacy under field conditions. Vaccine 15: 1101-1109. doi:10.1016/S0264-410X(97)00006-6. PubMed: 9269053. 9269053 PMC7131199

[3] Pedersen NC (2009) A review of feline infectious peritonitis virus infection: 1963-2008. J Feline Med Surg 11: 225-258. doi:10.1016/j.jfms.2008.09.008. PubMed: 19254859. 19254859 PMC7129802

[4] Hartmann K, Ritz S (2008) Treatment of cats with feline infectious peritonitis. Vet Immunol Immunopathol 123: 172-175. doi:10.1016/j.vetimm.2008.01.026. PubMed: 18395801. 18395801 PMC7132371

[5] Haagmans BL, Osterhaus AD (2006) Coronaviruses and their therapy. Antiviral Res 71: 397-403. doi:10.1016/j.antiviral.2006.05.019. PubMed: 16837072. 16837072 PMC7114240

[6] Hsieh LE, Lin CN, Su BL, Jan TR, Chen CM et al. (2010) Synergistic antiviral effect of Galanthus nivalis agglutinin and nelfinavir against feline coronavirus. Antiviral Res 88: 25-30. doi:10.1016/j.antiviral.2010.06.010. PubMed: 20603153. 20603153 PMC7114315

[7] Weiss RC, Toivio-Kinnucan M (1988) Inhibition of feline infectious peritonitis virus replication by recombinant human leukocyte (alpha) interferon and feline fibroblastic (beta). Interferon - Am J Vet Res 49: 1329-1335. 3178028

[8] McDonagh P, Sheehy PA, Norris JM (2011) In vitro inhibition of feline coronavirus replication by small interfering RNAs. Vet Microbiol 150: 220-229. doi:10.1016/j.vetmic.2011.01.023. PubMed: 21367541. 21367541 PMC7117188

[9] Regan AD, Shraybman R, Cohen RD, Whittaker GR (2008) Differential role for low pH and cathepsin-mediated cleavage of the viral spike protein during entry of serotype II feline coronaviruses. Vet Microbiol 132: 235-248. doi:10.1016/j.vetmic.2008.05.019. PubMed: 18606506. 18606506 PMC2588466

[10] van der Meer FJ, de Haan CA, Schuurman NM, Haijema BJ, Verheije MH et al. (2007) The carbohydrate-binding plant lectins and the non-peptidic antibiotic pradimicin A target the glycans of the coronavirus envelope glycoproteins. J Antimicrob Chemother 60: 741-749. doi:10.1093/jac/dkm301. PubMed: 17704516. 17704516 PMC7110056

[11] Balzarini J, Keyaerts E, Vijgen L, Vandermeer F, Stevens M et al. (2006) Pyridine N-oxide derivatives are inhibitory to the human SARS and feline infectious peritonitis coronavirus in cell culture. J Antimicrob Chemother 57: 472-481. doi:10.1093/jac/dki481. PubMed: 16387746. 16387746 PMC7110042

[12] Ballesteros ML, Sánchez CM, Enjuanes L (1997) Two amino acid changes at the N-terminus of transmissible gastroenteritis coronavirus spike protein result in the loss of enteric tropism. Virology 227: 378-388. doi:10.1006/viro.1996.8344. PubMed: 9018137. 9018137 PMC7130969

[13] Eckert DM, Kim PS (2001) Mechanisms of viral membrane fusion and its inhibition. Annu Rev Biochem 70: 777-810. doi:10.1146/annurev.biochem.70.1.777. PubMed: 11395423. 11395423

[14] Weissenhorn W, Dessen A, Calder LJ, Harrison SC, Skehel JJ et al. (1999) Structural basis for membrane fusion by enveloped viruses. Mol Membr Biol 16: 3-9. doi:10.1080/096876899294706. PubMed: 10332732. 10332732

[15] Kielian M, Rey FA (2006) Virus membrane-fusion proteins: more than one way to make a hairpin. Nat Rev Microbiol 4: 67-76. doi:10.1038/nrmicro1326. PubMed: 16357862. 16357862 PMC7097298

[16] Liu IJ, Kao CL, Hsieh SC, Wey MT, Kan LS et al. (2009) Identification of a minimal peptide derived from heptad repeat (HR) 2 of spike protein of SARS-CoV and combination of HR1-derived peptides as fusion inhibitors. Antiviral Res 81: 82-87. doi:10.1016/j.antiviral.2008.10.001. PubMed: 18983873. 18983873 PMC7114320

[17] Liu SW, Xiao GF, Chen YB, He YX, Niu JK et al. (2004) Interaction between heptad repeat 1 and 2 regions in spike protein of SARS-associated coronavirus: implications for virus fusogenic mechanism and identification of fusion inhibitors. Lancet 363: 938-947. doi:10.1016/S0140-6736(04)15788-7. PubMed: 15043961. 15043961 PMC7140173

[18] Yuan K, Yi L, Chen J, Qu X, Qing T et al. (2004) Suppression of SARS-CoV entry by peptides corresponding to heptad regions on spike glycoprotein. Biochem Biophys Res Commun 319: 746-752. doi:10.1016/j.bbrc.2004.05.046. PubMed: 15184046. 15184046 PMC7111000

[19] Bosch BJ, Martina BE, Van Der Zee R, Lepault J, Haijema BJ et al. (2004) Severe acute respiratory syndrome coronavirus (SARS-CoV) infection inhibition using spike protein heptad repeat-derived peptides. Proc Natl Acad Sci U S A 101: 8455-8460. doi:10.1073/pnas.0400576101. PubMed: 15150417. 15150417 PMC420415

[20] Henkel J (1999) Attacking AIDS with a 'cocktail' therapy? FDA Consum 33: 12-17. PubMed: 10443176. 10443176

[21] Ho DD (1995) Time to hit HIV, early and hard. N Engl J Med 333: 450-451. doi:10.1056/NEJM199508173330710. PubMed: 7616996. 7616996

[22] McHutchison JG, Gordon SC, Schiff ER, Shiffman ML, Lee WM et al. (1998) Interferon alfa-2b alone or in combination with ribavirin as initial treatment for chronic hepatitis C. Hepatitis Interventional Therapy Group. N Engl J Med 339: 1485-1492. doi:10.1056/NEJM199811193392101. PubMed: 9819446. 9819446

[23] Pedersen NC, Boyle JF, Floyd K (1981) Infection studies in kittens, using feline infectious peritonitis virus propagated in cell culture. Am J Vet Res 42: 363-367. PubMed: 6267959. 6267959

[24] Vogel L, Van der Lubben M, te Lintelo EG, Bekker CP, Geerts T et al. (2010) Pathogenic characteristics of persistent feline enteric coronavirus infection in cats. Vet Res 41: 71. doi:10.1051/vetres/2010043. PubMed: 20663472. 20663472 PMC2939696

[25] Lin CN, Su BL, Wu CW, Hsieh LN, Chueh LL (2009) Isolation and identification of a novel feline coronavirus from a kitten with naturally occurring feline infectious peritonitis in Taiwan. Taiwan Vet J 35: 145.

[26] Bosch BJ, van der Zee R, de Haan CA, Rottier PJ (2003) The coronavirus spike protein is a class I virus fusion protein: structural and functional characterization of the fusion core complex. J Virol 77: 8801-8811. doi:10.1128/JVI.77.16.8801-8811.2003. PubMed: 12885899. 12885899 PMC167208

[27] Porotto M, Yokoyama CC, Palermo LM, Mungall B, Aljofan M et al. (2010) Viral entry inhibitors targeted to the membrane site of action. J Virol 84: 6760-6768. doi:10.1128/JVI.00135-10. PubMed: 20357085. 20357085 PMC2903269

[28] Medinas RJ, Lambert DM, Tompkins WA (2002) C-Terminal gp40 peptide analogs inhibit feline immunodeficiency virus: cell fusion and virus spread. J Virol 76: 9079-9086. doi:10.1128/JVI.76.18.9079-9086.2002. PubMed: 12186891. 12186891 PMC136458

[29] Miller EH, Harrison JS, Radoshitzky SR, Higgins CD, Chi X et al. (2011) Inhibition of Ebola virus entry by a C-peptide targeted to endosomes. J Biol Chem 286: 15854-15861. doi:10.1074/jbc.M110.207084. PubMed: 21454542. 21454542 PMC3091195

[30] Lambert DM, Barney S, Lambert AL, Guthrie K, Medinas R et al. (1996) Peptides from conserved regions of paramyxovirus fusion (F) proteins are potent inhibitors of viral fusion. Proc Natl Acad Sci U S A 93: 2186-2191. doi:10.1073/pnas.93.5.2186. PubMed: 8700906. 8700906 PMC39932

[31] Wild CT, Shugars DC, Greenwell TK, McDanal CB, Matthews TJ (1994) Peptides corresponding to a predictive alpha-helical domain of human immunodeficiency virus type 1 gp41 are potent inhibitors of virus infection. Proc Natl Acad Sci U S A 91: 9770-9774. doi:10.1073/pnas.91.21.9770. PubMed: 7937889. 7937889 PMC44898

[32] Root MJ, Kay MS, Kim PS (2001) Protein design of an HIV-1 entry inhibitor. Science 291: 884-888. doi:10.1126/science.1057453. PubMed: 11229405. 11229405

[33] Kilby JM, Hopkins S, Venetta TM, DiMassimo B, Cloud GA et al. (1998) Potent suppression of HIV-1 replication in humans by T-20, a peptide inhibitor of gp41-mediated virus entry. Nat Med 4: 1302-1307. doi:10.1038/3293. PubMed: 9809555. 9809555

[34] Jiang S, Lin K, Strick N, Neurath AR (1993) HIV-1 inhibition by a peptide. Nature 365: 113. doi:10.1038/365113a0. PubMed: 8371754. 8371754

[35] He Y, Xiao Y, Song H, Liang Q, Ju D et al. (2008) Design and evaluation of sifuvirtide, a novel HIV-1 fusion inhibitor. J Biol Chem 283: 11126-11134. doi:10.1074/jbc.M800200200. PubMed: 18303020. 18303020

[36] Pan C, Lu H, Qi Z, Jiang S (2009) Synergistic efficacy of combination of enfuvirtide and sifuvirtide, the first- and next-generation HIV-fusion inhibitors. AIDS 23: 639-641. doi:10.1097/QAD.0b013e328325a4cd. PubMed: 19242316. 19242316 PMC2695507

[37] Pang W, Tam SC, Zheng YT (2009) Current peptide HIV type-1 fusion inhibitors. Antivir Chem Chemother 20: 1-18. doi:10.3851/IMP1369. PubMed: 19794228. 19794228

[38] Xu Y, Liu Y, Lou Z, Qin L, Li X et al. (2004) Structural basis for coronavirus-mediated membrane fusion. Crystal structure of mouse hepatitis virus spike protein fusion core. J Biol Chem 279: 30514-30522. doi:10.1074/jbc.M403760200. PubMed: 15123674. 15123674 PMC7982547

[39] Watanabe S, Takada A, Watanabe T, Ito H, Kida H et al. (2000) Functional importance of the coiled-coil of the Ebola virus glycoprotein. J Virol 74: 10194-10201. doi:10.1128/JVI.74.21.10194-10201.2000. PubMed: 11024148. 11024148 PMC102058

[40] Weiss RC, Oostrom-Ram T (1989) Inhibitory effects of ribavirin alone or combined with human alpha interferon on feline infectious peritonitis virus replication in vitro. Vet Microbiol 20: 255-265. doi:10.1016/0378-1135(89)90049-7. PubMed: 2549687. 2549687 PMC7117261

[41] Weiss RC, Cox NR, Boudreaux MK (1993) Toxicologic effects of ribavirin in cats. J Vet Pharmacol Ther 16: 301-316. doi:10.1111/j.1365-2885.1993.tb00177.x. PubMed: 8230401. 8230401 PMC7167023

[42] Ball RA, Kinchelow T, Group ISRS (2003) Injection site reactions with the HIV-1 fusion inhibitor enfuvirtide. J; Am Acad Dermatol 49: 826-831. 14576660 10.1016/s0190-9622(03)02099-1

[43] Kilby JM, Lalezari JP, Eron JJ, Carlson M, Cohen C, et al. (2002) The safety, plasma pharmacokinetics, and antiviral activity of subcutaneous enfuvirtide (T-20), a peptide inhibitor of gp41-mediated virus fusion, in HIV-infected adults. AIDS Res Hum Retroviruses 18: 685-693. 12167274 10.1089/088922202760072294

